# Hydrogels Based on Dynamic Covalent and Non Covalent Bonds: A Chemistry Perspective

**DOI:** 10.3390/gels4010021

**Published:** 2018-03-08

**Authors:** Francesco Picchioni, Henky Muljana

**Affiliations:** 1 Department of Chemical Engineering, Engineering and Technology Institute Groningen (ENTEG), University of Groningen, Nijenborgh 4, 9747 AG Groningen, The Netherlands; henky@unpar.ac.id; 2 Department of Chemical Engineering, Parahyangan Catholic University, Ciumbuleuit 94, Bandung 40141, West Java, Indonesia

**Keywords:** hydrogels, dynamic covalent bonds, reversible polymeric network

## Abstract

Hydrogels based on reversible covalent bonds represent an attractive topic for research at both academic and industrial level. While the concept of reversible covalent bonds dates back a few decades, novel developments continue to appear in the general research area of gels and especially hydrogels. The reversible character of the bonds, when translated at the general level of the polymeric network, allows reversible interaction with substrates as well as responsiveness to variety of external stimuli (e.g., self-healing). These represent crucial characteristics in applications such as drug delivery and, more generally, in the biomedical world. Furthermore, the several possible choices that can be made in terms of reversible interactions generate an almost endless number of possibilities in terms of final product structure and properties. In the present work, we aim at reviewing the latest developments in this field (i.e., the last five years) by focusing on the chemistry of the systems at hand. As such, this should allow molecular designers to develop a toolbox for the synthesis of new systems with tailored properties for a given application.

## 1. Introduction

In the last few years, a renewed interest in hydrogels has arisen due to an extended range of applications [1]. This stems from the characteristics of these materials such as biocompatibility and responsiveness to a variety of external stimuli [2]. While “classical” applications such as their use as an adsorbent in waste water treatment [3,4] are still heavily investigated, other areas are gaining significant attention at the moment. In particular, biomedical applications are very popular and include cell culture [5], wound dressing and healing [2,6], drug delivery [2,7,8], tissue engineering scaffolds [9], bone repair [10], and cartilage regeneration [11]. Such wide variety of biomedical applications stems from the peculiar characteristic of hydrogels, namely the fact that they can retain a large amount of water in their structure [1]. This makes it, in turn, also possible to incorporate water-soluble moieties in the final product such as proteins and DNA [5], but also dispersible (nano)particles [12,13] and (nano)emulsions [14]. 

**Figure 1:**
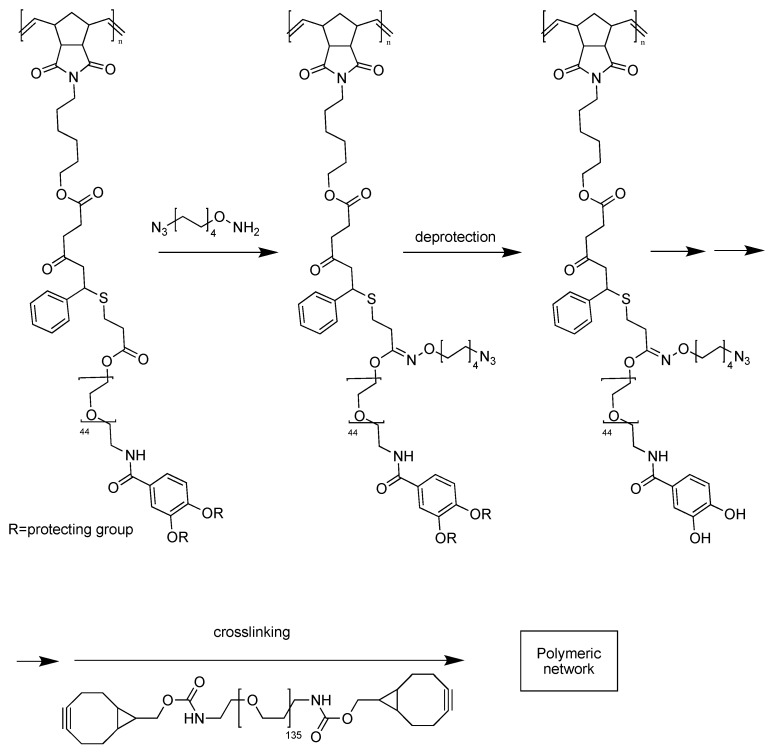
Incorporation of catechol groups in a polymeric network. Adapted and re-drawn from [15].

Particularly important is the adhesion at the interface between the polymeric chains and several different substrates to be then released (where and when needed) upon action of an external stimulus. On a molecular level, this translates in the presence of reversible bonds between the polymeric network and such “loading.” For example, the catechol chemistry (Figure 1) can actually be employed to ensure adhesion of the polymeric chains to several different moieties while crosslinking the network in an irreversible way [15].

In this particular case, the crosslinking via metal free click chemistry is practically quantitative and does not result in the formation of any by-product, and this rendering it ideal for biomedical applications [16,17,18]. 

The catechol chemistry is not the only example of a reversible yet covalent bond that can be formed and broken on command upon an external stimulus. Indeed, along the same strategy, the imine formation chemistry can be used to tune the adhesion [18].

The molecular reversibility can be actually achieved in two different ways: either by making use of equilibrium reactions (e.g., the Diels-Alder one) or through dynamic exchange reactions (e.g., reaction of an excess amino groups with epoxide ones). Both approaches have been widely used for tuning the adhesion of different substrates on different polymeric networks and for the network formations (and disruption) itself. These are discussed in the following paragraph.

## 2. Dynamic Hydrogels Based on Reversible (Covalent) Interactions

**Figure 2:**
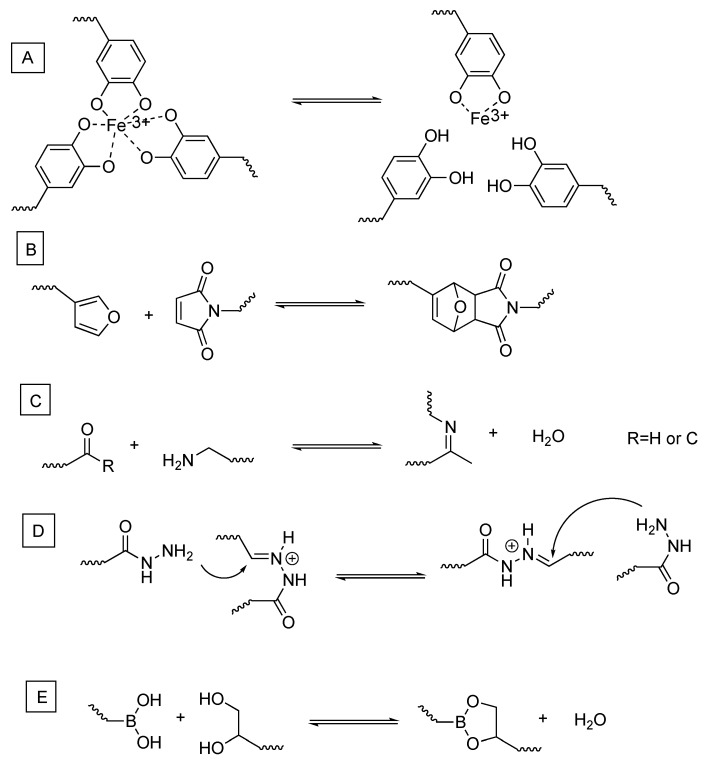
Schematic representation of reversible covalent bonds. (**A**): catechol-Fe chemistry; (**B**): furan-maleimide as example of cycloaddition reactions; (**C**): imine/enamine formation; (**D**): dynamic acylhydrazone exchange reaction; (**E**): boric ester formation and hydrolysis.

The same concept of covalent reversible bonds can be used for the hydrogel formation and disruption. Examples of interactions used in this case are (Figure 2) electrostatic ones [19,20], cycloadditions [8,21], redox reactions [22,23,24], and other ones such as imine [25,26,27,28] and enamine formation [29], acylhydrazone [30,31,32,33], and borax acid reaction with hydroxyls [13,34,35,36]. 

The general idea is that the use of dynamic covalent bonds allows the polymeric network to adjust itself as a result of an external stimulus. This can be achieved in principle through other weaker interactions, e.g., hydrogen bonding. In particular, a clear trend is detected in the last year, according to which self-assembly driven processes [10,37] can be conveniently used for hydrogel preparation. However, the use of covalent bonds displays two distinct and clear advantages [25]. In first instance, the network is still covalently linked, which renders it quite robust against small random fluctuations in environmental conditions such as temperature. Furthermore, exchange reactions such as the one of an amine with an imine are often kinetically controlled by the use of catalysts. In turn, this allows the possibility to freeze the network conformation (by slowing the kinetics) when desired.

**Figure 3:**
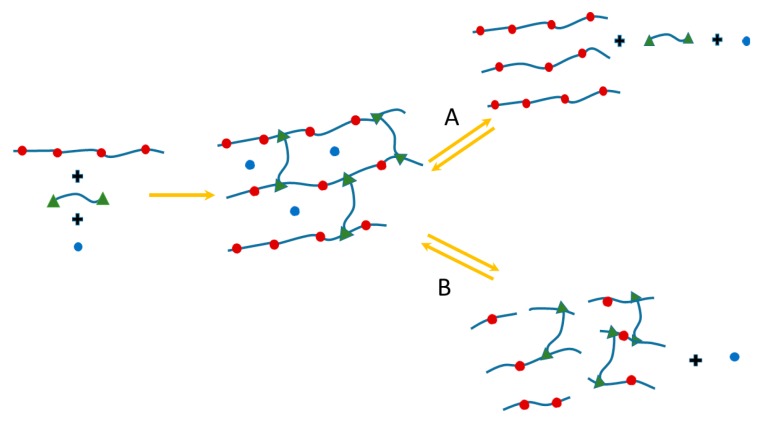
Schematic diagram for the strategy behind the use of reversible covalent bonds in hydrogels. (●) = reversible bonds along the backbone; (∆) = reversible bonds as crosslinking points; (●) = loading of the network (substrate).

The general concept behind the use of reversible interactions for the hydrogel polymeric chains is the (reversible) network disruption with immediate release of any loading (Figure 3).

Reversible bonds can be incorporated along the backbone (red circles) or at the crosslinking point (green triangles). The network, when subjected to an appropriate external stimulus, can then break at the crosslinking point (route A) or along the backbone (route B). This generates network fragments that can be quite different in terms of chemical structure even if in both cases the loading (blue circles) will be released. As a result of the network disruption, the load is released as the polymeric chains become soluble and not able anymore to entrap the load. In the specific case of drug delivery, this mechanism entails a controlled release rate depending on the kinetics of the network disruption, which in turn can be linked, at the molecular level, to the kinetics of the reversible bonds. 

**Figure 4:**
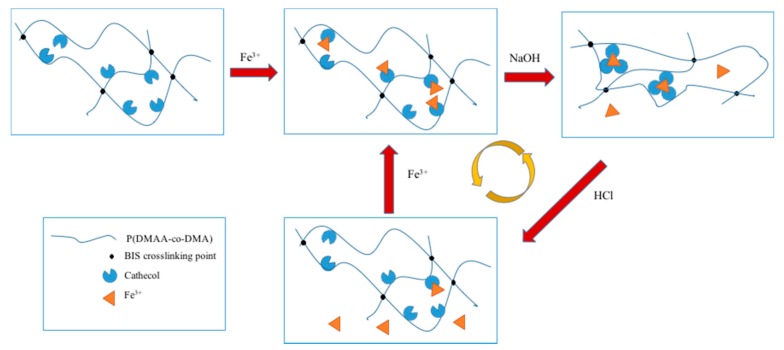
Schematic drawing of reversible complexation of DMA with Fe^3+^ ions. Adapted and re-drawn from [38] with permission.

An interesting example is based, once more, on the catechol chemistry [38] for which a hydrogel containing di-methylacrylamide units (DMA) can be reversibly crosslinked by reversible complexation with Fe^3+^ ions (see Figure 4).

In this particular case, the reversibility is cleverly achieved by switching between a tri- and a mono-chelate complex based on the pH of the solution. The corresponding final product displays a shape memory effect triggered by the pH value.

**Figure 5:**
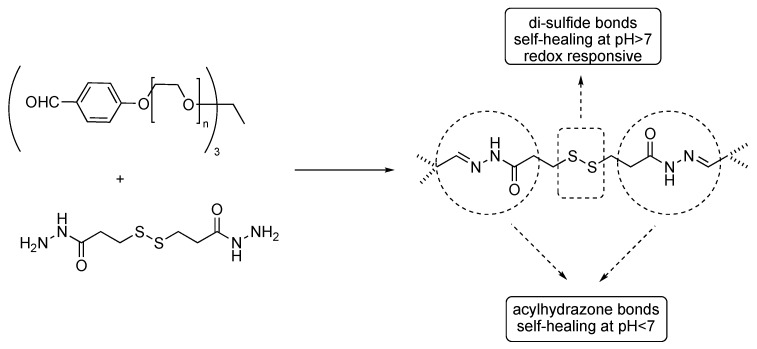
Dynamic hydrogels based on di-sulfide and acylhydrazone chemistry. Adapted and re-drawn from [39].

In order to achieve a sol-gel transition and, possibly, self-healing properties in response to multiple stimuli, different functional groups might be embedded along the backbone, e.g., di-sulfide and acylhydrazone ones, as shown in Figure 5 [39].

**Figure 6:**
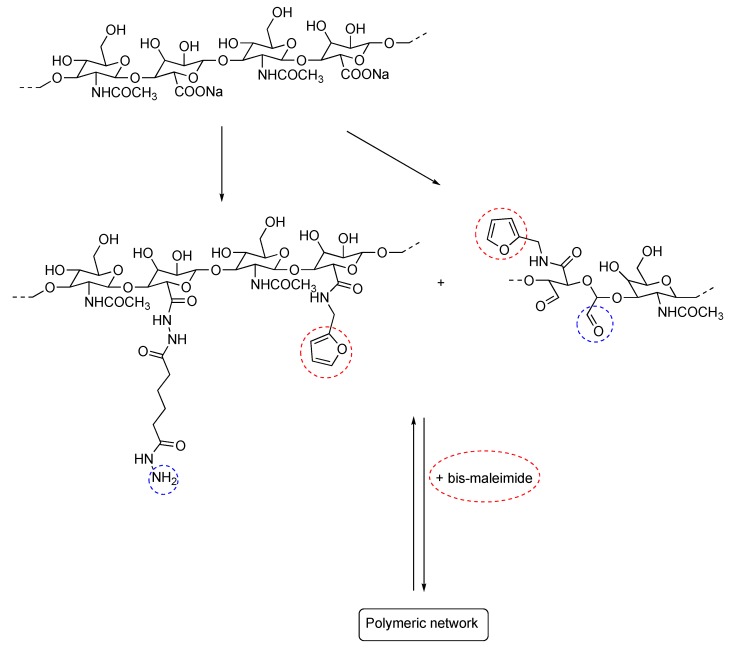
Double cross link network based on Diels-Alder and acylhydrazone reversible chemistry. Adapted and re-drawn from [9].

The advantage of such approach is that different functional groups are factually responsible for the response under different environmental conditions. Similar to this, hydrogels have been prepared for which both Diels-Alder and acylhydrazone groups are present along the backbone (Figure 6) [9].

**Figure 7:**
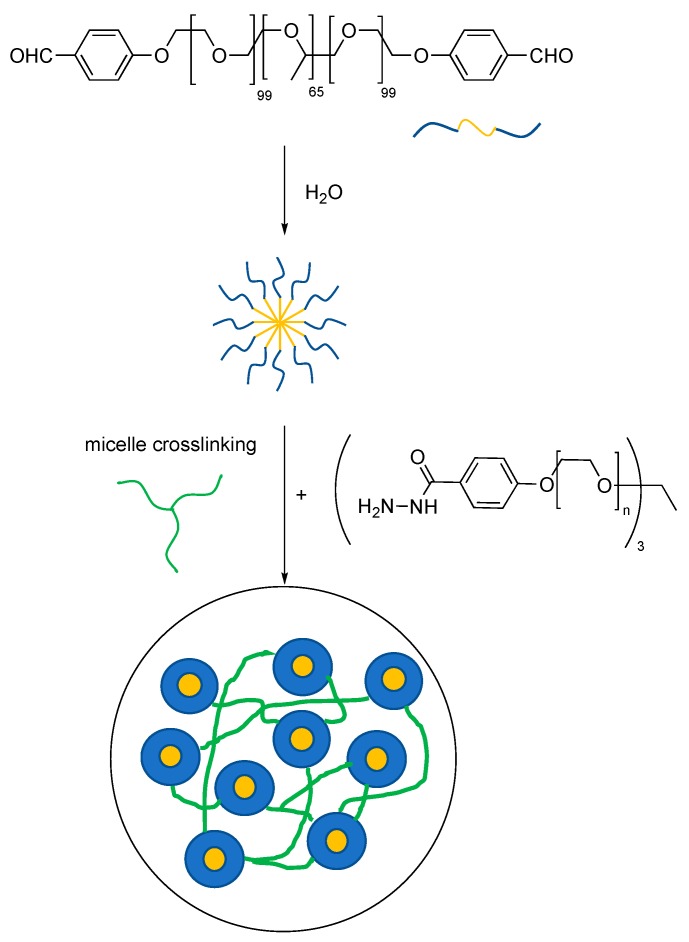
Dynamic covalent hydrogels with triblock copolymer micellization. Adapted and re-drawn from [33]. Yellow: hydrophobic block and/or domain. Blue: hydrophilic block and/or domain. Green: crosslinker.

In this case, it has been proposed that the Diels-Alder adducts preserve the gel integrity and endow it with good mechanical properties while the acylhydrazone reversible chemistry is conveniently employed to fine-tune the crosslinking density. The latter is in turn responsible for the self-healing behavior. The same hydrazone reversible chemistry can be conveniently combined with self-assembly of block copolymers to yield (see Figure 7) hydrogels with improved mechanical properties [33].

The resulting hydrogels still display self-healing properties and elongations up to 10.000%, the latter being a function of the pH. This stems from the dependency of the crosslinking density (acylhydrazone bonds) on the pH values. Such outstanding mechanical behavior (i.e., strain values) can also be attributed to the presence of the micelles, which actually act as physical crosslinking points for the all system.

**Figure 8:**
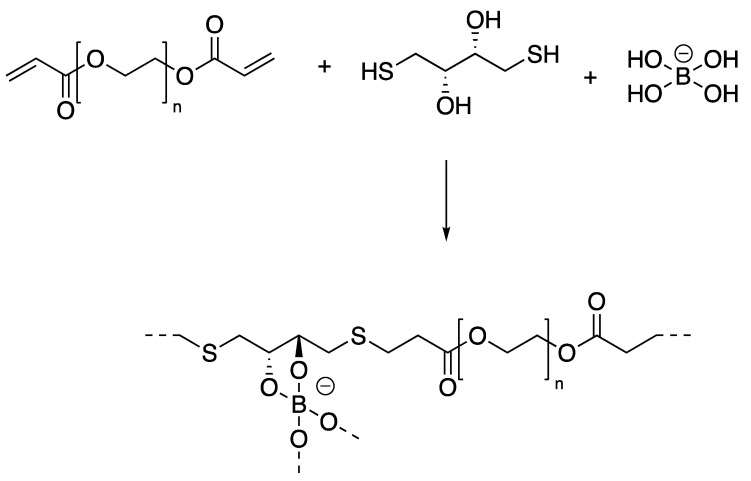
Hydrogels based on poly(ethylene-glycol) (PEG) with the use of thiol-ene addition and a borax-diol chemistry. Adapted and re-drawn from [35].

This approach, i.e., the use of two different reversible covalent bonding, represents a very popular choice in the last five years [40,41]. This stems from the fact that, besides the synergy in terms of reversible behavior as result of external stimuli and of combination of different properties, an additional one can be pursued in terms of the synthetic approach. A paradigmatic example is represented by the synthesis of hydrogels based on poly(ethylene-glycol) (PEG) with the use of thiol-ene addition as well as borax-diol chemistry (Figure 8) [35].

Borax acts in this case as catalyst for the thiol-ene reaction, thus factually helping in building the polymeric backbone, but also as crosslinking agent via the reversible borax-diol chemistry. The reversibility of the crosslinking was demonstrated by self-healing experiments and confirmed by rheology measurements. As typical for these systems, i.e., covalently and reversibly crosslinked gels, a cross over point between the elastic (G′) and loss (G″) modulus is observed for relatively moderate frequency values (1 < ω < 100 rad/s). This is in fact a very general characterization technique and observation [35] that can be used to characterize the response of the hydrogel to shear force.

**Figure 9:**
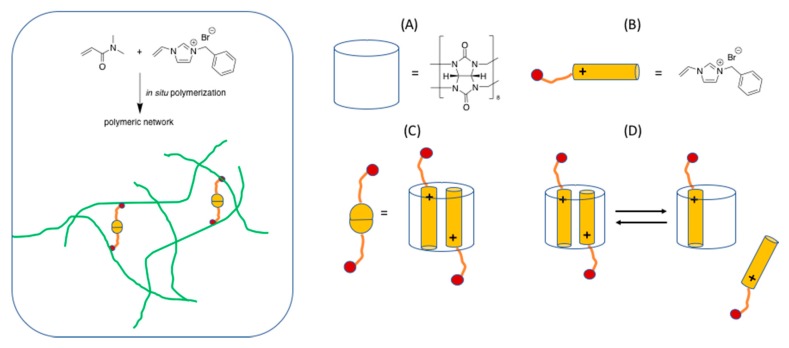
Adapted and re-drawn from [43]. Insert: green denotes the polymeric network. Yellow/orange structure: see (**B**,**C**). (**A**) chemical structure of host (**B**) chemical structure (**C**) host guest coupling (**D**) dynamic equilibrium for reversible crosslinking. Di-methylacrylamide is used here as an example of a monomer that can be used.

Very recently host-guest chemistry (molecular recognition) has been also reported for the synthesis of hydrogels [42,43]. In one example the proposed approach is rather versatile as it relies on fixed molecular recognition interaction even if in different matrixes prepared by in situ polymerization, as illustrated in Figure 9 [43].

The versatility of this method and its compatibility with several different monomeric precursors and polymeric end-products render it particularly attractive.

An interesting consequence of the dynamic character of these bonds is the fact that this significantly favors the adhesion between hydrogels and organo-gels (when in contact with each other), provided that such reaction can take place at the interface between these two gels [44].

A shown above, the choice of covalent reversible bonds for the network formation endows the final product with a multifaceted portfolio of properties (i.e., multiple responses to multiple stimuli). This can be achieved on the basis of several different substrates. Indeed, synthetic polymers are still widely used for the preparation of hydrogels, with polyacrylamide representing the most popular choice [45]. On the other hand, hydrogels based on natural products (e.g., chitosan [4,17], proteins [11,31,36], modified alginate [46], peptides [28,47]) represent a very convenient choice in view of clear advantages related to biological applications, but also to general sustainability principles. Indeed, attention is being paid also to novel synthetic pathways in agreement with green chemistry principles [48].

**Figure 10:**
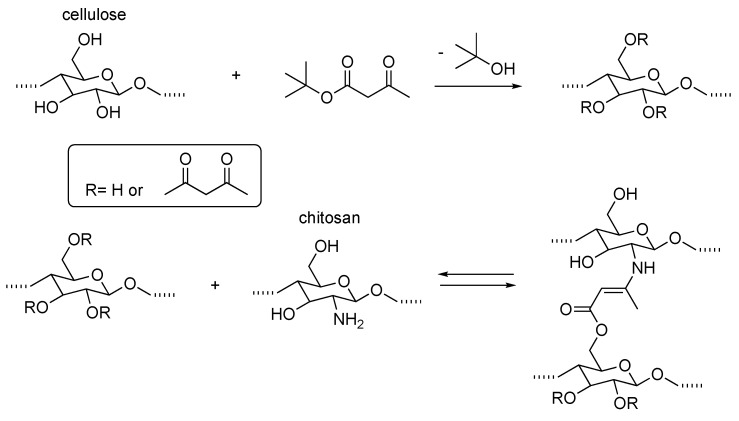
Polysaccharide hydrogels based on covalent enamine bond. Adapted and re-drawn from [29].

The choice of PEG as the main constituent of the polymeric backbone is an obvious one [49,50] when making allowances for the commercial availability of many (functionally modified) PEG varieties as well as their well-known thickening effect even in the presence of salts. On the other hand, polysaccharides also represent a popular choice mainly in view of the easiness of the modification and their availability in nature. An elegant example is the combination of modified cellulose with chitosan (Figure 10) [29].

The presence of an amino group along the chitosan chain is exploited here in order to achieve high reactivity (even at room temperature) with modified cellulose through enamine formation, in this case or via imine formation in other reported examples [51,52]. A combination of a natural polysaccharide with PEG combines the best of both worlds and has been recently reported [53].

## 3. Conclusions and Future Perspectives

As clearly seen form the example discussed above, the use of reversible covalent bonds in hydrogels endows the final product with very peculiar chemical structures, which in turns translate into a kaleidoscopic ensemble of possibilities in terms final properties. As also observed in other chemistry-related research fields, the study of synergistic phenomena seems to represent a very popular trend. In the specific case of hydrogels, this translates into the combination of multiple reversible interactions (and more specifically, reversible covalent bonds) in the same final product. This allows control of the network structure in processes such as self-healing in response to several different external stimuli. This is particularly attractive for biomedical applications where physiological parameters (e.g., pH, temperature, shear stress, etc.) might change in a simultaneous manner. On a molecular level, this generates the need for synthetic strategies allowing the incorporation of such reversible covalent bonds on the polymeric network. The above review of the most recent trends clearly show on one side the high sophistication level of the synthetic strategies employed and the multifaceted properties toolbox achievable in this way.

It must be stressed here that such research trends will probably continue in the coming years as the number of possibilities in terms of chemical bonds is certainly not yet exhausted. On the other hand, it is also conceivable that, as the topic will reach more scientific maturity, more attention will be paid to industrially feasible preparation routes. In this context, the choice of suitable substrates (i.e., polymeric materials) as well as synthetic strategies will probably constitute a focal point of future research projects.
